# Health literacy: setting an international collaborative research agenda

**DOI:** 10.1186/1471-2296-10-51

**Published:** 2009-07-10

**Authors:** Joanne Protheroe, Lorraine S Wallace, Gillian Rowlands, Jennifer E DeVoe

**Affiliations:** 1 NPCRDC, University of Manchester, 5th floor Williamson Building, Oxford Road, Manchester M13 9PL, UK; 2 University of Tennessee Graduate School of Medicine, Department of Family Medicine, 1924 Alcoa Hwy, U-67, Knoxville, TN 37920, USA; 3 Institute of Primary Care and Public Health, Department of Health GP Health Literacy Champion, Institute of Primary Care and Public Health, Faculty of Health and Social Care, London South Bank University, 103 Borough Road, London SE1 0AA, UK; 4 Department of Family Medicine, Oregon Health and Science University, 3181 Sam Jackson Park Rd, FM, Portland, OR 97239, USA

## Abstract

**Background:**

Health literacy is an increasingly important topic in both the policy and research agendas of many countries. During the recent 36^th ^Annual Meeting of the North American Primary Care Research Group, the authors led an audio-taped 3-hour forum, "*Studying Health Literacy: Developing an International Collaboration*," where the current state of health literacy (HL) in the United States (US) and United Kingdom (UK) was presented and attendees were encouraged to debate a future research agenda.

**Discussion of Forum Themes:**

The debate centred around three distinct themes, including: (1) refining HL definitions and conceptual models, (2) HL measurement and assessment tools, and (3) developing a collaborative international research agenda. The attendees agreed that future research should be theoretically grounded and conceptual models employed in studies should be explicit to allow for international comparisons to be drawn.

**Summary and Authors Reflections:**

The importance of HL research and its possible contribution to health disparities is becoming increasingly recognised internationally. International collaborations and comparative studies could illuminate some of the possible determinants of disparities, and also possibly provide a vehicle to examine other research questions of interest.

## Background

Health literacy is a topic that has been gaining momentum over the past few years, both in the policy and research agendas of many countries. During the 36^th ^Annual Meeting of the North American Primary Care Research Group, the authors led a 3-hour forum, "*Studying Health Literacy: Developing an International Collaboration*," where the current state of health literacy (HL) in the United States (US) and United Kingdom (UK) was presented. Interested participants at the conference were invited to attend, no preparation was required. Following a brief presentation 10 forum attendees, from the US, UK, and Canada, were encouraged to share their ideas–*blue-sky thinking*–about proposed research collaborations and the direction of future international research agendas. The discussion was facilitated by three of the authors (JP, LW and JD), but the direction of the discussion was led by the attendees. The attendees represented a range of academic and clinical disciplines, including health service research, nursing, medical and psychology and all had an expressed research interest in Health Literacy, individuals' extent of research experience was not known. Following approval from both the Institutional Review Board of the University of Tennessee Graduate School of Medicine-Knoxville and attendees, the forum was audio-recorded in its entirety to allow the authors to capture all content discussed. Author's handwritten notes and the audio recordings were referred to in order to present a summary of what was discussed, and as a result propose a direction for future international research in health literacy. We have attempted to capture all the topics discussed.

As described below, content discussed throughout the forum centred around three distinct themes, including: (1) refining health literacy definitions and conceptual models, (2) health literacy measurement and assessment tools, and (3) developing a collaborative international research agenda.

## Discussion of Forum Themes

### Refining Health Literacy Definitions and Conceptual Models

Forum attendees were eager to discuss their opinions regarding the scope and completeness of current health literacy definitions. For example, the *US Institute of Medicine *defines health literacy as,

"the degree to which individuals have the capacity to obtain, process, and understand basic information and services needed to make appropriate decisions regarding their health."[1]

In Europe, health literacy has been described similarly, but with a slightly wider emphasis, as

"the ability to make sound health decisions at home, in the community, workplace, healthcare system, marketplace, political arena. It is a critical empowerment strategy to increase people's control over their health, their ability to seek out information and their ability to take responsibility." [2]

There was a strong consensus among attendees that current health literacy definitions are limited in both scope and complexity. Attendees, representing various academic disciplines, noted that currently available health literacy definitions seem to consider abilities, skill and functions as equivalent. However, as expressed by a clinical psychologist, these entities are inherently different. For example, an individual might have adequate or good cognitive ability, including verbal skills (e.g., vocabulary, comprehension) and non-verbal skills (e.g., short-term memory, processing), which may be used to acquire knowledge and skills in an educational programme, but may have more difficulty in gathering information through inter-personal interactions. Given this, the group felt that it may be better to consider health literacy in terms of competencies, which could include such variables as knowledge, skills, abilities, and attitudes. Notwithstanding, health literacy definitions and perhaps competencies need to be revised to capture all related and relevant components.

While attempts at health literacy conceptualisation have been made [3-5], it was felt that there is a need to refine these models to line up with evolving health literacy definitions and competencies. Furthermore, researchers should take care to design their studies using health literacy conceptual models alone or in combination with established health behavior theories and models (e.g., Health Belief Model, Theory of Reasoned Action). Importantly, such conceptual and theoretical models should be adequately described in publications and/or presentations to (1) enable other researchers to replicate employed methodology more easily and (2) allow for parallel comparisons between studies across different patient or population-based populations. Future research should be theoretically grounded, and this should be explicit in future publications. Though this was not noted in discussion, this would be in parallel to the use of conceptual models and theories when interventions aimed at improving self-management are described in papers [6].

### Health Literacy Measurement and Assessment Tools

Over the past two decades a collection of predominantly English language health literacy assessment tools have been developed with the Rapid Estimate of Adult Literacy in Medicine (REALM) used most frequently [7]. While the limitations of various health literacy assessment tools has been compared and contrasted, collectively these tools are a more precise marker of health-related knowledge and health outcomes than is self-reported educational attainment [7]. We reached a group consensus that none of the currently available measures could be considered an ideal tool and that that current health literacy assessment tools will need to be redesigned, or new ones developed, to reflect revisions in health literacy definitions and accompanying conceptual models. It was felt that currently available tools did not adequately capture all aspects of the definitions of health literacy.

Attendees indicated a need to further refine health literacy assessment tools for use in diverse populations. As a starting point, the REALM was recently validated in a UK population [8]. As an exemplar, the International Physical Activity Questionnaire (IPAQ) was developed and validated to enable researchers to measure self-reported physical activity in a uniform manner across countries [9]. Parallel to the IPAQ, developing a multi-lingual health literacy assessment tool would allow for broader comparisons across diverse populations and countries.

### Developing an International Collaborative Research Agenda

Describing the detailed current state of health literacy research across the globe was beyond the scope of our forum and this commentary; we focused on English-speaking countries, with some specific examples from the US, Canada and the UK in developing some potential directions for collaboration, including:

• *Examining the Association between Health Literacy and Health Disparities*: Despite vastly different health care systems, significant health disparities exist within the developed world. Therefore, it would be interesting to explore the extent to which health literacy is associated with and/or possibly influences health disparities. Specifically, it would be interesting to not only explore how health literacy affects health disparities, but to determine if the larger healthcare system structures play a role in differing effects across countries. For example, the UK has the National Health Service and good general access to a comprehensive system of primary care, while in the US, access to healthcare is highly dependent upon insurance type or lack of insurance. Therefore, it would be interesting to examine if and how a person's health literacy influences access to needed medical care and uptake of preventive health measures.

• *Comparing the Link between Literacy and Health Outcomes across Countries*: As an example, both the US (*National Assessment of Adult Literacy–NAAL*) and the UK (*Skills for Life–SfL*) have conducted nationally representative literacy assessments in their populations [10,11]. However, current NAAL and SfL data are not linked with health-related data (e.g., health behaviors, access to needed healthcare services, health-related outcomes). Therefore, there is a need to marry these data in population-based studies. Furthermore, use of psychometrically sound international health literacy assessment tools in national literacy studies will be essential in making accurate direct country-to-country comparisons.

• *Exploring how Individuals in Different Countries Access Health-Related Information (e.g., Internet, media, healthcare professionals) as a Function of their Health Literacy Skills*: Specifically, does place of residence – for example, US versus UK–influence how health-related information is obtained as a function of one's health literacy skills? There is evidence that only a small portion of those falling into the 'below basic health literacy' designation on the NAAL reported receiving any type of health-related information [10]. It would be interesting to examine this question in the UK population as well.

• *Testing Educational Tools and Models*: Each individual country has models and tools being used to varying degrees to train and educate health professionals with differing educational levels and levels of training (e.g., physicians, nurses, pharmacists, phlebotomists) and ancillary staff (e.g., medical receptionists, certified medical assistants) about health literacy. However, there is a need for research to determine how best to share these models and to adapt these models to best fit local and regional circumstances.

## Summary and authors' reflections

In summary, the importance of health literacy research and its possible contribution to health disparities is becoming increasingly recognised internationally. The main points discussed at this forum were (i) the need to refine health literacy definitions and conceptual models; (ii) the need to refine health literacy measurement tools to be in line with definitions and conceptual models and (iii) the benefits of increased international collaborative research.

Further, we believe that research to attempt to untangle the potential effects of health literacy from poverty and other measures of social disadvantage would be valuable. Alongside linking data on literacy and health outcomes in population data, there is also a need to examine what effect, if any, would interventions designed to improve health literacy, have on health outcomes. In addition, or alongside the research agenda described above, health literacy might provide an ideal vehicle in which to examine other research topics of interest, for example the relatively poor health statistics (eg, life expectancy, maternal mortality rate) in the US compared to other wealthy industrialised countries, which appear to exist at all points of the socioeconomic distribution [12]. Furthermore, by exploring relative health literacy, and testing interventions designed to improve health literacy in differing health systems, some light may be shed on the possible benefits that might come from establishing a Medical Home Model in the US [13].

The next steps would be for researchers interested in the field of health literacy to consolidate current thinking and to build on these ideas to develop some truly stimulating and invigorating international research collaborations.

## Competing interests

The authors declare that they have no competing interests.

## Authors' contributions

All the authors devised the forum, JP, LW and JD led the three-hour forum JP conceived the idea of the article, and LW and JP wrote the early drafts. All authors read and contributed to the final manuscript.

## Pre-publication history

The pre-publication history for this paper can be accessed here:


